# Systematic review and meta-analysis of complications and mortality of veno-venous extracorporeal membrane oxygenation for refractory acute respiratory distress syndrome

**DOI:** 10.1186/s13613-017-0275-4

**Published:** 2017-05-12

**Authors:** Sergi Vaquer, Candelaria de Haro, Paula Peruga, Joan Carles Oliva, Antonio Artigas

**Affiliations:** 1 Critical Care Center, CIBER de Enfermedades Respiratorias, Corporació Sanitària Universitària Parc Taulí, Autonomous University of Barcelona, Parc Taulí 1, 08208 Sabadell, Spain; 2 Fundació Parc Taulí, Parc Taulí University Institute, Parc Taulí 1, 08208 Sabadell, Spain

**Keywords:** Extracorporeal life support (ECLS), Extracorporeal CO_2_ removal (ECCO_2_R), Acute respiratory failure (ARF), Mechanical ventilation, H1N1, Extracorporeal bypass

## Abstract

Veno-venous extracorporeal membrane oxygenation (ECMO) for refractory acute respiratory distress syndrome (ARDS) is a rapidly expanding technique. We performed a systematic review and meta-analysis of the most recent literature to analyse complications and hospital mortality associated with this technique. Using the PRISMA guidelines for systematic reviews and meta-analysis, MEDLINE and EMBASE were systematically searched for studies reporting complications and hospital mortality of adult patients receiving veno-venous ECMO for severe and refractory ARDS. Studies were screened for low bias risk and assessed for study size effect. Meta-analytic pooled estimation of study variables was performed using a weighted random effects model for study size. Models with potential moderators were explored using random effects meta-regression. Twelve studies fulfilled inclusion criteria, representing a population of 1042 patients with refractory ARDS. Pooled mortality at hospital discharge was 37.7% (CI 95% = 31.8–44.1; *I* ^2^ = 74.2%). Adjusted mortality including one imputable missing study was 39.3% (CI 95% = 33.1–45.9). Meta-regression model combining patient age, year of study realization, mechanical ventilation (MV) days and prone positioning before veno-venous ECMO was associated with hospital mortality (*p* < 0.001; *R* ^2^ = 0.80). Patient age (*b* = 0.053; *p* = 0.01) and maximum cannula size during treatment (*b* = −0.075; *p* = 0.008) were also independently associated with mortality. Studies reporting H1N1 patients presented inferior hospital mortality (24.8 vs 40.6%; *p* = 0.027). Complication rate was 40.2% (CI 95% = 25.8–56.5), being bleeding the most frequent 29.3% (CI 95% = 20.8–39.6). Mortality due to complications was 6.9% (CI 95% = 4.1–11.2). Mechanical complications were present in 10.9% of cases (CI 95% = 4.7–23.5), being oxygenator failure the most prevalent (12.8%; CI 95% = 7.1–21.7). Despite initial severity, significant portion of patients treated with veno-venous ECMO survive hospital discharge. Patient age, H1N1-ARDS and cannula size are independently associated with hospital mortality. Combined effect of patient age, year of study realization, MV days and prone positioning before veno-venous ECMO influence patient outcome, and although medical complications are frequent, their impact on mortality is limited.

**Electronic supplementary material:**

The online version of this article (doi:10.1186/s13613-017-0275-4) contains supplementary material, which is available to authorized users.

## Background

Despite extensive research and improved clinical management, mortality of severe forms of acute respiratory distress syndrome (ARDS) remains high [1–3]. However, in most cases, no specific treatment exists but to provide supportive therapy while the initial decompensating factor is treated. In this context, mechanical ventilation (MV) is cornerstone; however, ventilator-induced lung injury (VILI) remains a principal problem, with an impact on patient outcome [4]. Furthermore, despite best evidence-based respiratory care, a percentage of patients still die from refractory ARDS [5]. Therefore, in patients presenting with severe ARDS who become unresponsive to conventional treatment, the use of extracorporeal membrane oxygenation (ECMO), able to temporary replace pulmonary function, can represent a life-saving alternative, while time is gained for resolving the underlying cause [6].

ECMO can be used as a rescue therapy to avoid injurious effects of mechanical ventilation and to rescue from extreme gasometrical alterations [6]. These objectives can be attained by means of veno-venous ECMO circuits and can be used in patients with a large spectrum of underlying pathologies leading to refractory ARDS. New technologies with easier and more reliable equipment and procedures have allowed the expansion of veno-venous ECMO to many intensive care units (ICU) worldwide [7], and recent evidence suggested a potential positive effect of the use of these systems in refractory ARDS [8] as compared with initial experiences [9, 10]. However, despite the increasing number of reports, an adequate estimation of the effects of veno-venous ECMO use on complication rate and patient outcome using a meta-analytical approach is still lacking. Available studies provide either a general overview of a large historical database without pooled analysis [11], included high percentage of patients requiring cardiovascular support [12], are based on a specific population of patients [13] or were based on a low number of reports representing a small population of patients [14]. Therefore, we performed a systematic review and meta-analysis of the latest available peer-reviewed published literature to adequately estimate complication rate and hospital mortality associated with veno-venous ECMO in patients presenting refractory ARDS.

## Methods

The MEDLINE database from the National Library of Medicine (USA) and EMBASE were systematically searched to find articles reporting the utilization of veno-venous ECMO for treating any form of severe and refractory ARDS. Studies were included if they reported medical or technical complications in adult patients (>18 years old) from 1972 to December 2015. The search string was comprised of the following MESH terms and Booleans: ((“Extracorporeal Membrane Oxygenation”[Mesh] OR “Oxygenators, Membrane”[Mesh]) AND (“Respiratory Distress Syndrome, Adult”[Mesh] OR “Respiratory Insufficiency”[Mesh])) OR ((“Extracorporeal Membrane Oxygenation”[Mesh] OR “Oxygenators, Membrane”[Mesh]) AND “Respiratory Insufficiency”[Mesh] AND (“Carbon Dioxide”[Mesh] OR “Hypercapnia”[Mesh])) AND “humans”[MeSH Terms] AND (“adolescent”[MeSH Terms] OR “adult”[MeSH Terms]). The term “Oxygenators, Membrane” was added to include older articles classified before 1988. The term “adolescent” was included to permit allocation of studies reporting patients ageing 18 years old (PubMed search engine identifies an “adult” as a 19 years or more subject). This implied the inclusion of a number of paediatric cases ageing >16 years, who had to be later excluded from the analysis. Additional articles were allocated by reviewing article reference lists, textbooks and grey literature.

Articles retrieved were included or rejected based on information obtained from the title, abstract or main text. Only studies reporting the use of veno-venous ECMO techniques were included. In studies reporting a combination of veno-venous ECMO and veno-arterial ECMO, only patients who received VV-ECMO were included if information about patient outcome and technique complications could be differentiated for each technique. In all other cases, studies were included if the percentage of VA-ECMO utilization was below 10%. This percentage was arbitrarily selected on the basis of its expected negligible effect on the statistical analysis and to account for the reduced percentage of patients that present associated cardiac failure to refractory ARDS. Meta-analysis and systematic reviews were excluded to avoid overlapping of results with original articles. Articles reporting analysis from the international ELSO—Extracorporeal Life Support Organization—were also excluded to avoid overlapping of patients with those presented in articles published by the original submitting centre. Only articles written in English were included in this systematic review.

A minimum number of 50 veno-venous ECMO cases per report were arbitrarily established as an initial threshold to minimize publication bias. Risk of bias of studies was further assessed using a scoring system for evaluating selection, attrition and reporting biases for randomized controlled trials (max. 3 points) [15] and the Ottawa–Newcastle scale for non-randomized studies (max. 9 points) [16]. Studies were identified to have high risk of bias and were excluded if their scoring results were below 3 and 5 points, respectively, or if information on patient outcome and complications was not provided. Additionally, small study effect was evaluated by means of visual evaluation of funnel plots and the Egger’s test of the intercept, using mortality at discharge from hospital as the main outcome variable and a random effects model analysis, with significant threshold at one-tailed *p* < 10% as originally described [17]. Duval and Tweedie’s Trim and Fill method [18] was used to impute potentially missing studies on both sides of the plot using a random effects model and to compute corrected mean point estimates if required.

Three investigators reviewed all articles, performed data extraction and complied the database using equal digital templates (VS, dHC, PP). Results were compared, and discrepancies were solved by agreement. A fourth investigator approved the final database and decided upon remaining conflictive data (AA). In case of suspected unreliable information due to insufficient clarity in data presentation, the article was excluded from the analysis. Any other source of conflict was solved by consensus of the research team (VS, dHC, PP, OJ, AA). The process followed the PRISMA guidelines for systematic reviews and meta-analyses [19] and the Cochrane guidelines for systematic review of interventions [20].

Once appropriate articles were allocated, the following general study and technical information were collected: type of study, centre and country of realization, year of study start and year of finalization, number and cause of veno-venous ECMO (ARDS, lung transplant, trauma, hypoventilation, mixed), patient age, pre-ECMO PO_2_/FiO_2_ ratio (P/F), positive end-expiratory pressure and plateau pressure (Pplat), Sequential Organ Failure Assessment Score (SOFA) [21] if available, MV days before ECMO initiation, Lung Injury Score (LIS) [22], centre experience (determined by the number of veno-venous ECMO cases per year), veno-venous ECMO duration, cannulation site (jugulo-femoral or J-F, femoro-jugular or F-J, femoro-femoral or F-F, J double, F double or mixed), maximum cannula size used (in French), membrane type (silicone—spiral, polymethylpentene/polypropylene—hollow fibre), pump type (rotary, centrifugal), mean measured or targeted coagulation time ratio during ECMO treatment, total number of complications, mortality attributable to complications and mortality at hospital discharge. Patient deceases were considered to be attributable to complications of veno-venous ECMO whenever authors of the original article clearly identified a direct relation between the occurrence of the complication and the fatal outcome. Such complication must have occurred during the course of veno-venous ECMO treatment or immediately after its discontinuation. The presented average coagulation time during ECMO in each article was divided by the standard laboratory time of the specific test if provided, or by 107 s for activated coagulation time (ACT), 35 s for partial thromboplastin time (PTT) and 22 s for thrombin time (TT) to obtain a comparable coagulation ratio across studies. Studies were sorted according to their finalization and start year to allow evaluation of the impact of study realization time on study variables. If such information was not available, one year before acceptance for publication was arbitrarily selected as the study finalization year.

The following medical complications were recorded: total bleeding events, significant bleeding events (defined as major/severe bleeding by the authors of the original article due to relevant impact on patient status, bleeding requiring surgical control or haemoderivate transfusion and bleeding events leading to fatal outcome), number of specific site bleeding events (surgical site, respiratory system, gastrointestinal, cannulation site, intracerebral, haemothorax and other), pneumothorax (if related to cannulation only), limb ischaemia, diffuse intravascular coagulopathy, heparin-induced thrombocytopenia, haemolysis, deep venous thrombosis, pulmonary embolism, severe arrhythmia, hypothermia, confirmed bloodstream infection after veno-venous ECMO initiation, veno-venous ECMO catheter infection, tamponade or direct cardiac injury. The total number of haemoderivates administered was also recorded (packed red blood cell units—RBC; platelet + fresh frozen plasma concentrates—PL/FFP).

The following equipment complications were recorded: oxygenator failure (oedema, coagulation or rupture), cannulas failure (disconnection, coagulation or rupture), decannulation events, pump failure (coagulation or rupture) and tubing malfunction (rupture, leak or coagulation).

Statistical evaluation was performed using SPSS version 22 statistical software (International Business Machines. Armonk, NY, USA), Comprehensive Meta-Analysis version 3 (Biostat Inc. Englewood, NJ, USA) and OpenMetaAnalyst version 10.10 [23]. Meta-analytic pooled estimation of mortality at hospital discharge, complications and mortality due to veno-venous ECMO complications was performed using a weighted random effects model for study size. Results are presented as overall average point estimate (in  %) with 95% CI and usual statistics (mean, median, CI 95% and IQR). Heterogeneity values (*I* ^2^) are provided when appropriate. Meta-regression using a random effects model analysis was employed to explore the potential effect of moderators on study variables. The following variables were evaluated as potential independent moderators: percentage of prone positioning before extracorporeal support, patient age, P/F ratio, PEEP, Pplat, driving pressure, MV duration, LIS and SOFA before veno-venous ECMO, cause of ARDS, extracorporeal support duration, centre experience, realization year, targeted/measured coagulation ratio during treatment, maximum cannula size used and membrane and pump type used. Combinations of moderator variables were evaluated by means of a stepwise meta-regression analysis in a random effects model. Variables included in the model were selected to maximize study inclusion in the analysis according to completeness of the database.

## Results

**Fig. 1:**
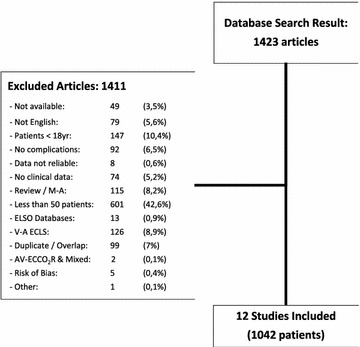
Consort chart of included/excluded studies

**Fig. 2:**
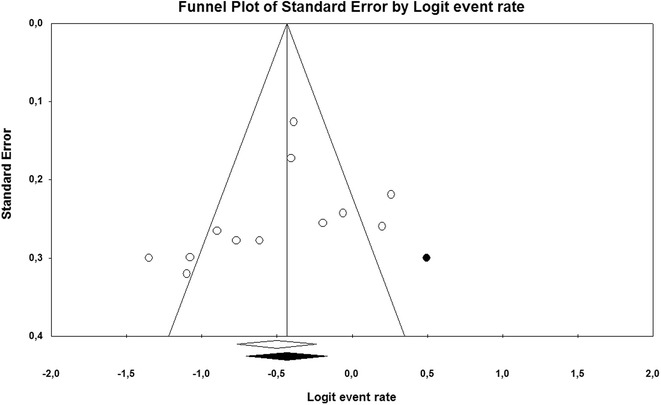
*Funnel plot* of included studies. *White circles* represent observed studies. Mean point was computed using a random effects model and is presented as white rhomboid. Using the Trim and Fill method, one additional imputable study was identified. The estimated corrected mean point with confidence interval is presented as a *black rhomboid*

**Fig. 3:**
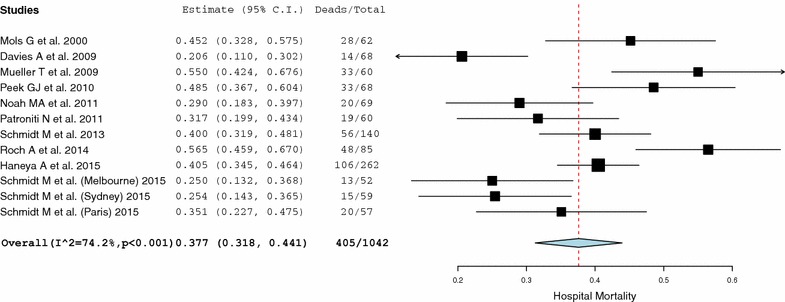
Forest plot—hospital mortality

The systematic search performed returned a total of 1423 studies. The complete list of studies can be found as Additional file 1: Study Database. One thousand four hundred eleven studies were excluded from the analysis based on exclusion criteria established for this systematic review and meta-analysis (Fig. 1). Main excluded studies are presented in Table 1. The study performed by Schmidt et al. [24] provided data from patients treated in three different centres from two nationalities. Since detailed information was provided for each of them, they were considered as independent studies and included separately to enhance statistical power of the analysis. The reports by Schmid et al. [25] and Camboni et al. [26] were excluded from the analysis to minimize the chance of patient overlap among studies performed in the same institution (University Medical Center, Regensburg, Germany). Main features of the final 12 included studies are presented in Table 2. All included studies were performed in the last fifteen years and used veno-venous ECMO for supporting patients under severe and refractory ARDS. Using hospital mortality as the main variable, included studies were evaluated for study size effect. The generated funnel plot presented no clear asymmetry upon visual inspection (Fig. 2), and the Egger’s test of the intercept did not identify any significant association between study size and hospital mortality (*t* = 1.23; df = 10; *p* = 0.12). Pooled mortality at hospital discharge was 37.7% (z = −3.73; CI 95% = 31.8–44.1%; *p* < 0.001) with a heterogeneity of *I* ^2^ = 74.2% (*Q* = 42.66; Tau^2^ = 0.153; *p* < 0.001) (Fig. 3). One imputable missing study was found using Duval and Tweedie’s Trim and Fill method on the right side of the funnel plot (Fig. 2). Corrected hospital mortality including the calculated missing study was 39.3% (CI 95% = 33.1–45.9%). The following moderator variables were found to be independently associated with hospital mortality: patient age (*b* = 0.053; *Q* = 6.6; *n* = 12; *p* = 0.01), maximum cannula size used during treatment (*b* = −0.075; *Q* = 7.04; *n* = 4; *p* = 0.008) and cause of ARDS (H1N1 = 24.8 vs other = 40.6%; *Q* = 4.894; *p* = 0.027). The meta-regression model combining patient age (*b* = 0.192; *p* < 0.001), year of study realization (*b* = −0.176; *p* = 0.003), MV days before veno-venous ECLS (*b* = 0.209; *p* = 0.003) and prone position before extracorporeal support (*b* = −2.383; *p* = 0.01) was associated with the observed hospital mortality (*Q* = 19.48; *n* = 11; *p* < 0.001; *R* ^2^ = 0.80). No additional moderator variables were associated with hospital mortality. Medical complications occurred in 40.2% of patients, being bleeding the most frequent (29.3%). Mechanical complications affected 11% of patients, with 12.8% of them requiring oxygenator replacement for malfunction. However, mortality caused by complications accounted for only 7% of deceases (Table 3).

**Table 1:** Main excluded studies

Study	Year	Patients	Reason for exclusion
Lehle et al. [42]	2014	317	No complications reported
Cheng et al. [43]	2013	216	Unable to identify data associated with veno-venous ECMO
Schmid et al. [44]	2012	176	Risk of patient overlap (duplicate)
Hemmila et al. [28]	2004	168	>10% veno-arterial ECMO
Bartlett et al. [45]	2000	146	>10% veno-arterial ECMO
Camboni et al. [26]	2011	127	Risk of patient overlap (duplicate)
Lindskov et al. [46]	2013	124	>10% veno-arterial ECMO
Pham et al. [35]	2013	123	>10% veno-arterial ECMO
Pranikoff et al. [47]	1999	94	Risk of bias
Bein et al. [48]	2006	90	Arterio-venous CO_2_ removal
Nehra et al. [49]	2009	81	Reports patients <18 years
Rubino et al. [50]	2014	72	Risk of bias
Liebold et al. [51]	2002	70	Reports patients <18 years
Chiu et al. [52]	2015	65	No complications reported
Pappalardo et al. [53]	2013	60	ELSO database
Ma et al. [54]	2012	56	Risk of bias
Chimot et al. [55]	2013	52	Risk of bias
Pranikoff et al. [56]	1994	51	>10% veno-arterial ECMO
Zimmermann et al. [57]	2009	51	Arterio-venous CO_2_ removal
Peek et al. [58]	1997	50	Reports patients <18 years

**Table 2:** List of included studies

Study	Year	Centre	Type	Patients	Population	P/F ratio	MV (days)	Prone (%)	LIS	Age (years)	Heparin coating	Membrane type	Pump type	Coagulation ratio^b^	Max cannula size (French)	Cannulation	ECMO duration (days)
Mols et al. [59]	2000	University of Freiburg, Freiburg, Germany	CP	62	Mixed	64	10	81	3.2	35	Y	n/a	R	1.26	25	F-J	14.5
Davies et al. [36]	2009	Multicentre, Australia	CR	68	H1N1	56	2	18	3.2	34^a^	Y	HF-PMP	C	n/a	n/a	Mixed	10^a^
Mueller et al. [60]	2009	University Medical Center, Regensburg, Germany	CR	60	Mixed	64	1	n/a	3.6	53^a^	Y	HF-PMP	C	1.50	23	F-J	9^a^
Peek et al. [8]	2010	The Glenfield Hospital, Leicester, UK	RCT	68	Mixed	70^a^	1.5^a^	47	3.5^a^	40	n/a	HF-PP	R	1.58	n/a	n/a	9^a^
Noah et al. [61]	2011	Multicentre, UK	CC	69	H1N1	55^a^	4^a^	39	3.5^a^	36	n/a	n/a	n/a	n/a	23	n/a	9^a^
Patroniti et al. [62]	2011	Multicentre, Italy	CP	60	ARDS	63^a^	2	27	3.6	41	Y	HF-PMP	C	1.6	n/a	Mixed	9^a^
Schmidt et al. [27]	2013	Multicentre, France	CR	140	Mixed	53	5	61	n/a	44^a^	Y	HF mixed	C	1.357	n/a	Mixed	15^a^
Roch et al. [63]	2014	Marseille North Hospital, France	CP	85	Mixed	60	2	20	3.5	47	Y	HF mixed	C	1.28	25	F-J	9
Haneya et al. [64]	2015	University Medical Center, Regensburg, Germany	CR	262	Mixed	64	1	n/a	3.3	49	Y	HF mixed	C	1.57	31	Mixed	9
Schmidt et al. [24] (Melbourne)	2015	Alfred Hospital, Australia	CR	52	Mixed	75	1	2	n/a	37	n/a	n/a	n/a	n/a	n/a	Mixed	10
Schmidt et al. [24] (Paris)	2015	Pitié-Salpètrière Hospital, France	CR	57	Mixed	61	4	20	n/a	46	n/a	n/a	n/a	n/a	n/a	Mixed	10
Schmidt et al. [24] (Sydney)	2015	Royal Prince Alfred Hospital, Australia	CR	59	Mixed	66	2	54	n/a	40	n/a	n/a	n/a	n/a	n/a	Mixed	9

*CP* cohort prospective, *CR* cohort retrospective, *CC* case–control, *RCT* randomized controlled trial, *P/F* ratio: PO_2_/FiO_2_ ratio, *MV* mechanical ventilation before ECMO initiation, *LIS* Lung Injury Score, *HF* hollow fibre, *PP* polypropylene, *PMP* polymethylpentene, *C* centrifugal, *R* rotatory, *F*-*J* femoro-jugular. Mean values are presented unless indicated

^a^Median values

^b^Normalized coagulation ratio, see “Methods” section for explanation

**Table 3:** Patient outcome and complication rate

	Number of studies reporting data	Average point estimate (CI 95%)
Hospital mortality	12	37.7% (31.8–44.1)
Mortality due to complications	8	6.9% (4.1–11.2)
Mortality due to bleeding	7	3.3% (2–5.4)
Medical complications	12	40.2% (25.8–56.5)
Bleeding	12	29.3% (20.8–39.6)
Significant bleeding	9	10.4% (5.6–18.7)
Cannula bleeding	8	9.3% (5.3–15.6)
ICH	5	5.4% (2.7–10.3)
Pulmonary bleeding	5	6.4% (3.2–12.4)
Other bleeding	6	9.3% (4.9–16.9)
DVT/PE	3	4.6% (2.2–9.2)
Pneumothorax	3	5.7% (1.1–24.2)
Cannula infections	3	9.9% (4.2–21.5)
Mechanical complications	4	10.9% (4.7–23.5)
Oxygenator failure	2	12.8% (7.1–21.7)
Cannula failure	3	4.5% (2.5–8.1)

*ICH* intracerebral haemorrhage, *DVT/PE* deep venous thrombosis/pulmonary embolism

## Discussion

Results of the present meta-analysis indicate that more than 60% of the patients with refractory ARDS who receive veno-venous ECMO survive hospital discharge despite initial high illness severity. These results are unexpected, given the observed mortality ratios in severe forms of ARDS [1–3]. Interestingly, Zampieri et al. [14] had already identified a potential beneficial effect of veno-venous ECMO on survival [14] in a meta-analysis of three studies including patients with severe and refractory ARDS (1 RCT and 2 case–control studies). However, reduction of hospital mortality could only be found if alternative severity-pairing method in two of the three included studies was used (OR = 0, 52; CI 95% = 0.35–0.76; *p* < 0.001). The low number of studies included representing a limited population of refractory ARDS patients may explain these results. Conversely, data from the present meta-analysis are supported by a larger number of studies, more recently published and by a larger population size of refractory ARDS patients receiving veno-venous ECMO. Moreover, thanks to the larger population being studied, details on technique-associated complications, their effect on patient outcome and the effect of potential moderator variables could be evaluated. Results of the present report are also consistent with those published in studies based on the Extracorporeal Life Support Organization (ELSO) database. The ELSO is an international association of ECMO-providing centres, which maintains a registry of ECMO cases and provides annual ECMO survival information and centre-based performance reports [7]. In the study by Paden et al., based on a large cohort of refractory ARDS patients receiving veno-venous ECMO, mortality rates oscillated between 36 and 49% depending on the condition leading to ARDS [7]. However, these results may be not completely representative of the true reality of ECMO use in ICUs worldwide. Selection and notification bias may affect studies based on the ELSO database, for that inclusion of patients into the registry is voluntary and only selected centres can be members of the network. Interestingly, two of the three studies included in the above-mentioned meta-analysis by Zampieri et al. were based on the ELSO database [14]. In this sense, the present report provides more reliable information by excluding studies from the ELSO database, which avoids above-mentioned biases and ensures minimum overlapping of patients and duplication of results. Finally, it also allowed us to gather more detailed information about procedural and centre characteristics not included in more general studies.

To explain the observed levels of hospital mortality found in the present meta-analysis and its associated heterogeneity, we explored the effect of potential moderator variables independently and in combination in a logistic meta-regression model. Patient age at study inclusion was independently associated with hospital mortality in our results, which confirm previous observations [27, 28] and is supportive of the validity of the database and the analysis performed. Such association was also found in the combined meta-regression model which suggests the principal role of this moderator variable. Indeed, patient age is a strong determinant of patient outcome that must be taken into account when ECMO treatment is indicated. Nevertheless, it is challenging to find a proper comparable population of patients with severe ARDS without ECMO to compare the specific effect of age on outcome. In the study by Guérin et al. [29], patients in the prone group had an average age of 58 years (compared to the present 42 years of ECMO patients determined in this meta-analysis) and presented inferior mortality rates (16 vs 39.3%). However, such patients probably had less severe lung injuries, not yet refractory to conventional treatment, including prone positioning. In the study by Guérin et al. [29], P/F ratio was substantially higher in the prone group than in patients included in the present meta-analysis (100 vs 62), and PEEP, Pplat and driving pressure values were higher in ECMO patients (14.2 vs 10; 32.6 vs 23; 7.6 vs 4 cmH_2_O, respectively). These findings indicate the need for higher ventilator loads to maintain minimum oxygenation, which would be caused by a more severe and established lung injury in veno-venous ECMO patients. Conversely, according to other reports published by Villar et al. [2] and Bellani et al. [3], higher mortality ratios in older patients with severe ARDS can be identified (58.1% and 46% vs 39.3%). It is therefore paramount that multiple factors are taken into account when trying to compare ECMO and non-ECMO patients, such as patient comorbidities and patient typology. In fact, the inclusion of an undetermined number of H1N1 patients in the present meta-analysis could have contributed to the observed association between mortality and patient age, given the younger average age of these patients. Other factors, such as immunocompromised status and malignancies, have the potential for significantly influencing outcome and are discussed later in this section.

Present results also suggest that cannula size used during veno-venous ECMO is also a determinant factor for patient outcome. Insufficient cannula size implies limited oxygenation capabilities of the extracorporeal system and consequently impossibilities the reduction of mechanical ventilation loads exerted on the patient during veno-venous ECMO treatment. As recently demonstrated, driving pressure is associated with patient outcome [30] and inability to reduce it during veno-venous ECMO has a negative influence on patient evolution [31]. Although insufficient information on driving pressure during extracorporeal support could be found to be included in the general meta-regression evaluation, the identified inverse association between cannula size and mortality could be indicative of the risks of insufficient extracorporeal support due to smaller-than-necessary cannula use. However, these results need to be evaluated cautiously, given the limited number of studies that reported the size of the used cannulas.

We were also able to find a statistically significant difference in hospital mortality in studies reporting predominantly H1N1 patients when compared to those in which veno-venous ECMO was initiated for a combination of causes leading to ARDS. This finding is also in accordance with previous observations, in which patients with viral pneumonia receiving ECMO presented inferior levels of mortality [7, 13]. These results are also not unexpected given the inferior mortality rates of H1N1 patients when compared with general ARDS population [32–34]. In another meta-analysis by Zangrillo et al. on mortality of H1N1 patients receiving veno-venous ECMO, authors estimated 28% (CI 95% = 18–37%) of pooled hospital mortality [13], which is similar to what has been observed in our results. However, high variability can be observed in results of studies evaluating the use of ECMO for severe and refractory ARDS induced by H1N1 infection. These oscillate between 17% in Schmidt et al. [27], 36% in Pham et al. [35] and 35% in Davies et al. [36]. Such variability could be caused by several factors ranging from pre-existing comorbidities to centre experience and patient management differences. As discussed below, the use of prone positioning before veno-venous ECMO is associated with improved outcomes. In the above-mentioned studies, the highest percentage of prone positioning can be observed in the study with the lowest mortality ratios (Schmidt et al. = 59%, Pham et al. = 45%, Davies et al. = 22%). In addition, the use of veno-arterial ECMO for concomitant cardiovascular failure in these studies was the lowest in the study with the lowest mortality ratios (5, 7.3 and 13%, respectively). As it will also be discussed later in this section, the use of veno-arterial ECMO implies exposure to a different pattern of associated complications, which have the potential for exerting an impact on patient outcome. Higher use of veno-arterial ECMO could also illustrate higher severity of patients, with added cardiovascular dysfunction, which would worsen prognosis and explain the different mortality rates.

Another variable, study realization time, was also associated with hospital mortality in the evaluated meta-regression model. Increasing centre ECMO experience during the studied time frame could be a factor explaining such association. Interestingly, centre experience has already been associated with improved outcomes in previous reports [37] but could not be identified as a moderator variable in the model used in the present analysis. Nevertheless, it is important to highlight that the used estimator of centre experience in our study does not take into account other forms of extracorporeal life support, which share technical and operational characteristics with veno-venous ECMO and could have been performed in the reporting centres during the same study time frame. Such additional extracorporeal support experience could not be incorporated into our analysis. Furthermore, other patient types that received veno-venous ECMO simultaneously but presented other pathologies not matching the objectives of the included studies were also not incorporated in the estimation of experience. This would be especially true for studies reporting only H1N1 patients. The effect of such underestimation of centre experience may be the cause of the observed lack of association with hospital mortality. Furthermore, the impossibility to ascertain centre experience in multicentre studies also limited the use of this variable in the combined meta-regression evaluation due to excessive missing information. On another side, progressive technological evolution of veno-venous ECMO equipment, with improved biocompatibility and reduced complication rates, could have had a potential positive impact on patient evolution [38]. Interestingly, previous reports have identified the opposite effect of study realization time in other forms of ECMO for cardiovascular support, in which mortality increased with publication year [12].

Mortality of refractory ARDS patients treated with veno-venous ECMO was also associated with MV days before extracorporeal support initiation in the combined meta-regression model. It has already been well established that a delayed instauration of ECMO and a prolonged MV before treatment (>7 days) have deleterious effects on outcome [28, 39]. Despite the fact that in most of the included studies veno-venous ECMO was initiated without much delay (median = 2, IQR = 1.1–4), we were able to identify an association of MV duration before extracorporeal support and mortality. This corroborates previous observations and justifies the initiation of veno-venous ECMO in refractory ARDS patients within the first days after admission, before irreversible lung injury is established.

Implementation of prone position before ECMO was also evaluated in the present report. Despite the already demonstrated beneficial effect of prone positioning in moderate–severe ARDS patients [29], prone positioning before veno-venous ECMO was only used in 40% of the included patients in the present systematic review and meta-analysis and presented high variability (range 2–73%). As recently published, prone position use is also very variable in patients with severe ARDS in ICUs worldwide [3]. In our results, prone positioning before veno-venous ECMO initiation was identified as a cofactor associated with positive outcomes. These results are also in accordance with those in previous studies [27]. In spite of the fact that in all included studies veno-venous ECMO was used in patients considered refractory to conventional treatment and followed pre-established protocols, which aimed at optimizing patients before treatment initiation, present results indicate that prone positioning was used insufficiently before veno-venous ECMO initiation. We were unable to ascertain whether such findings were due to excessive severity and instability of patients, or because other rescue therapies had been used instead of prone positioning. Limited information was available in this regard in the original studies included.

Finally, adequate patient selection of veno-venous ECMO treatment instauration also has the potential for influencing outcome [27, 28, 39] and may have also influenced results. Outcomes of ECMO treatment are highly dependent on the underlying cause of ARDS and patient comorbidities. In addition to already mentioned differences according to the cause of ARDS (H1N1 vs. other causes), patients with haematological malignancies and ARDS present markedly higher mortality ratios than those observed in present results (haematological = 50% present other = 40.6%, present H1N1 = 24.8%) [40]. Furthermore, patient immunocompromised status has already been identified as a prognostic factor in patients receiving veno-venous ECMO [27]. However, we were unable to allocate information on immune status or malignancy prevalence in the included studies to confirm previous observations.

Marked differences can be found in studies evaluating patients receiving ECMO for cardiovascular support when compared to ECMO for respiratory support. Veno-arterial ECMO has been associated with mortality rates that vary from 57%, in highly experienced centres [37], to 66% according to the ELSO database [7]. In these reports, patients received extracorporeal support for isolated cardiovascular failure mainly. Indeed, veno-arterial ECMO for cardiovascular failure must be differentiated from veno-venous ECMO for respiratory patients, for that it uses a different cannulation technique in which blood is directly reinjected into the arterial mainstream. This technique is also applied to a different type of patient with specific underlying pathology. Zangrillo et al. performed another meta-analysis to evaluate mortality rates and complications associated with ECMO use [12]. Studies included were developed in high case volume centres (>100 patients per study) and included patients who received support with veno-arterial ECMO for cardiac failure predominantly (92%). When such results are compared with present observations, marked differences in mortality at hospital discharge can be observed (39 vs 54%). Interestingly, Zangrillo et al. also identified a significant reduction in mortality rates in the subgroup of patients who only received veno-venous ECMO (*b* = −0.203; CI 95% = −0.412 to 0.005; *p* = 0.005). These results support that veno-arterial and veno-venous ECMO represent different techniques with different outcome rates. This important conceptual differentiation is of high relevance when designing a national ECMO programme for respiratory support, since both hospital resources and required professional expertise differ for both techniques. This also has a significant impact in cost estimation [8] and should be taken into account by healthcare administrators.

Data from our meta-analysis also indicate that, like in veno-arterial ECMO, medical complications associated with veno-venous ECMO are common. However, despite their high incidence during the treatment course, medical complications associated with ECMO support have a small impact in overall mortality, accounting only for 7% of fatal outcome cases. Additionally, although bleeding has been historically considered the most feared complication during ECMO, present results suggest that while bleeding episodes are rather frequent, their attributable mortality in veno-venous ECMO is reduced. Similarly, although catastrophic, ICH affects a limited number of patients. When present data are compared with results from the previous meta-analysis by Zangrillo et al., in which high rates of patients treated with veno-arterial ECMO were included [12], meaningful differences between the two techniques are once again noticeable. Bleeding episodes were inferior in the present report (33 vs 29%), so was the occurrence of intracerebral haemorrhage (8 vs 5.4%). Interestingly, limb ischaemia events were not reported in the included studies of the present meta-analysis. These findings illustrate the key differential features of veno-venous and veno-arterial ECMO circuits. The lack of arterial catheterization implies minimum risk of arterial ischaemia and also reduces the risk of arterial emboli and central nervous system complications. Furthermore, bleeding has been reported to be commonly observed in the cannula insertion site and in recent surgical wounds in either form of ECMO [7]. However, in the present report, surgical bleeding was reported only in a limited number of included studies. Since the likelihood of veno-arterial ECMO being performed close to cardiac surgery is higher, more frequent surgical site bleeding events should be expected in patients receiving this type of therapy. Also, the fact that cannulas are placed in a pressurized vessel may also predispose to increased bleeding. On the other side observed coagulation ratios in included studies with veno-venous ECMO were inferior to those attained in previously reported veno-arterial ECMO patients (mean = 1.45 vs 1.64) [12]; however, no association between coagulation ratio and increased bleeding rates could be observed in our results with veno-venous ECMO patients. It is necessary to comment that this lack of association may be caused by the insufficient validity of an average/target coagulation point that does not represent the true course of anticoagulation therapy. Oscillations of anticoagulation values not illustrated by mean values can occur during treatment and may have an impact on bleeding occurrence. Nevertheless, the possible benefits of veno-venous ECMO must be gauged to the lethality of the above-mentioned complications. This is specially true in case of ICH and other similar complications, which can have a significant impact on patient morbi-mortality and long-term effects on patient’s quality of life. Despite low attributable mortality to medical complications, emphasis must be put in adequate patient selection and experienced management of ECMO to further minimize their occurrence and impact on patient outcome.

Mechanical complications were present in 11% of patients. These findings contrast with historical reports in which high number of both oxygenator and pump failures were observed. However, two recent evaluations of complications associated with ECMO use in ARDS found that acute replacements of the ECMO system were required in 14.4 and 16.1% of cases [7, 41]. These results fall within the observed range of failures found in our meta-analysis. Nevertheless, the reduced number of studies reporting on technical and mechanical complications included in the present meta-analysis makes the interpretation of the above-mentioned results difficult and should be evaluated cautiously. Further and more technically oriented evaluations are required to provide a definitive answer on the technical improvement of veno-venous ECMO systems.

In addition to the already mentioned limitations of this study, some further aspects which may limit the interpretation of present results must be discussed. The highest statistical quality of systematic reviews and meta-analysis is obtained when randomized controlled trials (RCT) are used to estimate pooled outcomes [20]. Indeed, this minimizes the risk of multiple biases and enhances the quality of information obtained from the analysis. However, in the present report, the only recent RCT available on the use of ECMO for respiratory failure [8] was combined with non-randomized studies. In order to reduce bias in our results, strict inclusion criteria were implemented and careful care was taken to minimize the impact of confounding factors. In addition, specific statistical methods to identify publication bias and to quantify the impact of study size on results were implemented in the present report. None of the implemented evaluations suggested unreliability of present analyses, and results were adjusted to include potentially missing reports. Nevertheless, as new RCT become available, new meta-analyses should be performed to include newest data and improve reliability of present estimations. Another limitation of the present report is that not all studies provided information in all variables. While the main study variables were widely covered by most of the reports included, information on certain medical and technical complications was only provided in a few studies. Thus, the pooled point estimate for certain variables needs to be interpreted cautiously. Future studies will also be beneficial to provide better estimations of these variables. The present report is also limited by the fact that only articles written in English were included, which could represent a source of bias. However, the impact of such idiomatic bias was estimated to be negligible given the low number of reports written in other languages (5.6%). Finally, the high heterogeneity levels observed for hospital mortality rates implied increased variance among the included studies. Although we were able to identify one model that could explain a high proportion of the observed variance, additional unexplored factors can be present. The inclusion of data from upcoming RCTs, with more homogeneous patient characteristics, objectives and methodologies, may help in minimizing heterogeneity and yield more reliable and definitive results.

## Conclusion

Results suggest that despite high initial severity, patients treated with veno-venous ECMO for refractory ARDS present reduced mortality ratios. Patient age, H1N1-related ARDS and cannula size are independently associated with hospital mortality, and the combined effect of patient age, year of study realization, MV days and prone positioning before extracorporeal support are also associated with better outcomes. Medical complications are commonly present in veno-venous ECMO but have limited impact on patient outcome. When compared with veno-arterial ECMO for cardiovascular support, veno-venous ECMO presents a different pattern of complications and outcomes, probably due to differences in technical and patient characteristics.

In conclusion, veno-venous ECMO has evolved substantially and has become a widespread technique with progressively improving outcomes in recent years. Appropriate patient selection, timing and use of validated treatment options, including prone positioning before initiation of extracorporeal support, are key to treatment success. Despite the still relevant number of complications associated with veno-venous ECMO, attributable mortality is limited.

## Additional file

**Additional file 1.** Study Database

## Abbreviations

ARDS: acute respiratory distress syndrome
MV: mechanical ventilation
VILI: ventilation-induced lung injury
ECMO: extracorporeal membrane oxygenation
ECCO_2_R: extracorporeal CO_2_ removal
ICU: intensive care unit
ELSO: Extracorporeal Life Support Organization
P/F: PO_2_/FiO_2_ ratio
SOFA: Sequential Organ Failure Assessment Score
LIS: Lung Injury Score
J-F: jugulo-femoral
F-J: femoro-jugular
F-F: femoro-femoral
ACT: activated coagulation time
PTT: partial thromboplastin time
TT: thrombin time
RBC: red blood cell unit
PL/FFP: platelet + fresh frozen plasma concentrate
ICH: intracerebral haemorrhage
DVT/PE: deep venous thrombosis/pulmonary embolism
